# Perceived pressure to breastfeed negatively impacts postpartum mental health outcomes over time

**DOI:** 10.3389/fpubh.2024.1357965

**Published:** 2024-04-04

**Authors:** Rebecca E. Grattan, Sophie M. London, Georgia E. Bueno

**Affiliations:** School of Psychology, Te Herenga Waka, Victoria University of Wellington, Wellington, New Zealand

**Keywords:** postpartum mental health, maternal mental health, breastfeeding, breastfeeding intervention, mental health

## Abstract

**Introduction:**

Positive maternal mental health is associated with improved outcomes for infants, and yet the consideration of maternal mental health is often neglected in breastfeeding interventions. Breastfeeding interventions typically focus on breastfeeding promotion, and do not always include supports for the mother. This may result in isolated perceived pressure to breastfeed, the mental health impacts of which are not well understood.

**Methods:**

This mixed-methods, longitudinal study examined whether perceived pressure to breastfeed was associated with depression, suicide ideation, anxiety, birth trauma and stress concurrently and 4 weeks later for postpartum mothers. It also examined qualitative experiences of feeding.

**Results:**

Perceived pressure to breastfeed was associated with increased anxiety, stress and birth trauma symptoms four weeks later. Thematic analysis suggested this may be due to difficulties living up to the “breast is best” ideal, believing breastfeeding was part of success as a mother, lack of choices and autonomy in feeding choices for infants and general lack of support.

**Discussion:**

As such it appears we may be doing more harm than good by focusing our interventions for breastfeeding primarily on increasing pressure to breastfeed, and interventions should consider strategies for promoting positive maternal mental health alongside breastfeeding.

## Introduction

The favorable impacts of breastfeeding have been thoroughly examined, albeit in primarily correlational studies, demonstrating that breastfeeding is associated with preferred outcomes for infants including better cognitive functioning (1) and reduced infections (2). Successful breastfeeding journeys also provide mothers with feelings of accomplishment and connection (3). As such, low rates of breastfeeding remain a concern for international child health organizations (4, 5). However, current approaches aimed at increasing breastfeeding may lack a nuanced focus on maternal mental health, and the potential negative impacts of current intervention strategies on both the infant and mother have received little attention (6). Interventions aimed at increasing breastfeeding may inadvertently increase individual and societal pressure to successfully breastfeed, while lacking appropriate nuanced and individualized support for those struggling (6). As a result, these breastfeeding interventions may contribute to both negative maternal mental health, and also to breastfeeding difficulties through increasing maternal stress and guilt.

To increase rates of breastfeeding, one must consider what barriers to breastfeeding and prolonged breastfeeding exist. Barriers to breastfeeding appear to be complex, and differ by country and socioeconomic status, with many barriers existing at a societal level [e.g., public policy; (7)]. However, it appears some key barriers that are consistent across cultures include a lack of breastfeeding knowledge, maternal mental health difficulties, and breastfeeding difficulties (7). In terms of breastfeeding knowledge, it does appear the majority of mothers, even those from disadvantaged backgrounds, report to “know” breastfeeding is the best option for their baby (8). Therefore, it is likely there are reasons beyond a lack of breastfeeding knowledge that contribute to why women are not breastfeeding. However, findings do suggest that mothers who have inadequate knowledge about the *challenges* of breastfeeding are less likely to practice it (7). For example, women who had the belief that breastfeeding is “natural” and would not be difficult may be more likely to give up when problems occur during the breastfeeding process (9). This highlights the importance of comprehensive education about breastfeeding, that goes beyond promotion, and incorporates discussion about breastfeeding challenges and ways to handle them (9). In terms of breastfeeding difficulties, studies have found that difficulties are reported by up to 70.3% of mothers (10). Breastfeeding difficulties are diverse and can include pain when breastfeeding, supply issues, allergies or discomfort in the infant, excessive crying, and fatigue from breastfeeding (7). Importantly, challenges during breastfeeding are associated with early cessation of breastfeeding (11, 12) and negative maternal mental health symptoms (13). Finally, the relationship between maternal mental health difficulties and breastfeeding appears to be bi-directional. Maternal mental health difficulties are associated with lower rates of exclusive breastfeeding (14, 15), and fear and sadness experienced in relation to breastfeeding is associated with an increased likelihood of ceasing breastfeeding (16). Ending breastfeeding earlier than intended, and breastfeeding difficulties are both associated with negative mental health outcomes (17). Successful breastfeeding is associated with better mental health in mothers compared to those who do not breastfeed, which is commonly interpreted by researchers as breastfeeding leading to improved mental health [e.g., (18)]. However, this research is typically cross-sectional. Without being able to infer causality, it is possible that these findings indicate that the mother’s ability to successfully meet societal expectations of breastfeeding is what is supporting positive mental health.

Given concerns about low rates of breastfeeding, public health strategies aiming to increase rates of exclusive breastfeeding are prioritized (6), and there have been strategies put in place to reduce the promotion of breast-feeding alternatives such as formula feeding (19). For example, with the aim of combating large corporations that make money off breast-milk substitutes, there are guidelines to limit the promotion of formula such as ‘The International Code of Marketing of Breastmilk Substitutes’ (20). Breastfeeding promotion strategies vary significantly in both design and application. Common strategies to increase breastfeeding initiation and/or duration include large scale awareness programs (e.g., social media campaigns, prenatal education), one on one supports (e.g., breastfeeding support, counseling), and organization or community interventions [e.g., encouragement to breastfeed in hospitals, increasing breastfeeding friendly environments in hospitals; (21)]. Past taglines of international breastfeeding campaigns include “babies were born to be breastfed” in 2004 in the United States (22) and “breast is best” in the 1990s from the World Health Organization. The “breast is best” messaging appears to have been very influential with both breastfeeding and formula feeding mothers aware of this premise (23). Another prominent international strategy was ‘The Baby-Friendly Hospital Initiative’ which involves the promotion of breastfeeding via education and breastfeeding friendly practices within hospitals (24).

An unavoidable component of these breastfeeding interventions is that they involve, in some manner, increased pressure on mothers to breastfeed (25). For example, breastfeeding education typically involves educating mothers that infants experience better outcomes if breastfed, and it often does not include education on other feeding types or on breastfeeding difficulties. This education process may signal to mothers that formula feeding is wrong or bad, which reduces a woman’s ability to make an informed decision. The lack of discussion on breastfeeding difficulties can also set mothers up for disappointment with breastfeeding if issues arise. Within the Baby-Friendly Hospital Initiative, promotion of formula feeding is similarly discouraged, and a study in Scotland found that this initiative resulted in the restriction of discussion around non-breastfeeding infant feeding practices, leading to feelings of pressure to breastfeed and a lack of comprehensive feeding information (26). As such, despite best intentions, it appears many breastfeeding promotion strategies include a focus on pressure to breastfeed. This pressure may inadvertently cause shame, guilt and stress which could increase mental health problems in mothers, and as such, increase breastfeeding cessation. Particularly when mothers are experiencing difficulties breastfeeding. Further, these strategies may oppose a health consumers’ right to be fully informed and make decisions for themselves (26).

In support of this idea, breastfeeding promotion has been shown to elicit negative emotions for mothers (27). Further, interventions which are more focused on supporting mothers, such as counseling approaches, appear to have better success (28). There are also a limited number of qualitative studies that have indicated many mothers feel a strong pressure to breastfeed, which can lead to guilt and self-blame if they are not able to breastfeed (23). The impacts of pressure to breastfeed on maternal mental health have yet to be studied quantitatively, and longitudinally, with many groups calling for more research in the area (6, 25). Given maternal mental health is an important predictor of healthy child development (29), and increased stress can reduce breastfeeding capabilities (30), this is an important consideration.

This study aims to understand whether perceived pressure to breastfeed has a detrimental impact on maternal mental health, using a longitudinal mixed-methods approach in a New Zealand sample of mothers and other birthing parents. Depression, suicide ideation, anxiety, birth trauma, and stress outcomes were measured, alongside a qualitative examination of the experiences of those who experienced pressure to feed their infant in a certain way. Findings are discussed alongside suggestions for best practice in developing interventions that can improve breastfeeding rates and support positive maternal mental health.

## Methods

### Participants

An initial sample of 475 participants was gathered. Participants were excluded from the final sample if they were not between 6 weeks and 6 months postpartum (*n* = 24), if they did not complete more than 2 of the questionnaires at time 1 (*n* = 37), and if they did not complete the study (*n* = 189), leaving 225 eligible participants. Participants in the final sample included 225 postpartum mothers or birthing parents who were between 6 weeks to 6 months postpartum and were living in Aotearoa, New Zealand at the time of survey 1. This included 175 people who identified as NZ European (77.8%), 35 who identified as Māori (15.6%), 21 who identified as Asian (9.3%), 5 who identified as Pasifika (2.2%) and 5 who identified as other ethnicities (2.2%). Two participants did not identify as cisgender females. Mothers or birthing parents were aged from 20 to 44 (*M* = 30.20, SD = 4.67). Excluded participants did not differ from the included participants by whether or not they had a mental health history, nor whether or not they breastfed or experienced breastfeeding pressure. They were more likely to be Māori [*X*^2^ (1, *N* = 474) = 7.28, *p* < 0.01] but did not differ across other demographic variables. Excluded participants were more likely to report suicide ideation at time 1 [*X*^2^ (1, *N* = 460) = 5.44, *p* < 0.05], and had higher mean birth trauma [*t* (432.8) = −2.15, *p* = 0.03] and depression scores at time 1 [*t* (423.9) = −3.06, *p* < 0.01], but did not differ by other mental health symptoms.

### Procedure

Participants were recruited via widespread online social media advertising (Facebook and Instagram targeted advertising to parents of children under 12 months living in New Zealand), alongside posters put up in the community across New Zealand, and advertisements were also shared by groups relevant to new parents including Plunket New Zealand between November 2022 and November 2023. The advertisement was titled “Postpartum mental health survey.” Those interested were able to click a link, or use a QR code to sign up for the study, and were automatically emailed a link to the time 1 survey, and 4 weeks later were emailed a link to the time 2 survey. Participants completed the two self-report surveys approximately 4 weeks apart (actual time between survey completion of time 1 and time 2 ranged from 28 days to 116 days, *M* = 41.7, SD = 18.2) on Qualtrics.

### Measures

#### Breastfeeding and feeding measures

Perceived pressure to breastfeed, shame about infant feeding choice, stress related to infant feeding, whether or not someone breastfed, and reasons for choosing type of infant feeding were measured as part of the self-report demographic information at time 1. Each of these items were binary measures, experienced or not experienced. Participants who indicated that they did experience pressure to breastfeed, shame regarding breastfeeding, and/or stress around infant feeding to were asked to elaborate on their experiences, and these responses were used for the qualitative analysis.

#### Anxiety

Anxiety symptoms were measured at time 1 and time 2 using the Beck Anxiety Inventory (31), a reliable and valid measure of physical anxiety symptoms. Items were rated as how bothersome they were in the past month from 0 = *not at all* to 3 = *severely – it bothered me a lot*, and the sum score was used, which could range from 0 to 63.

#### Depression

Depression symptoms were measured at time 1 and time 2 using a modified version of the Edinburgh Postnatal Depression Scale (32). The scale was modified to reduce overlap with other measures, and two items related to anxiety were removed (“*I have been anxious or worried for no good reason*” and “*I have felt scared or panicky for no good reason*”). This left 8 items which are rated as how often they occurred in the past week from 0 = *never* to 3 = *quite often*, and total sum scores could range from 0 to 24.

#### Suicide ideation

Suicide ideation was measured at time 1 and time 2 using the Depressive Symptom Index-Suicidality Subscale (33). This subscale is a is a four-item self-report measure with good internal consistency and convergent validity (33). Items assess suicide ideation frequency, suicide ideation plans, suicide ideation controllability and suicide ideation impulses in the past 2 weeks. Each item includes a four-point scale with different anchors to indicate increasing severity. Higher scores indicate higher suicide ideation severity, and total scores range from 0 to 12. For the present analysis scores were converted to a dichotomous variable (0 = *no presence of ideation*, 1 *= presence of ideation*) given small reporting rates of suicide ideation in our final sample (*n* = 36).

#### Birth trauma symptoms

Birth trauma symptoms were measured at time 1 and time 2 using the City Birth Trauma Scale (34), which is a self-report measure of post-traumatic stress disorder symptoms following birth, with acceptable internal consistency and construct validity (34). This calculates a sum score from 22 PTSD symptom items rated on the following scale of how often the symptom occurred in the past week from 0 = *not at all*, 1 = *once*, 2 = *2–4 times*, 3 = *5 or more times*. Sum scores can range from 0 to 66.

#### Stress

Stress was measured at time 1 and time 2 using a shortened version of the 46-item version of the Maternal Postpartum Stress Scale (35). This is a self-report measure of experiences of typical postpartum stressors in the past month. A condensed list of 30 items was created with duplicate items condensed to one item. For example, “*Owing money*” and “*Financial difficulties*” were reduced to one item “*Financial difficulties*” to reduce participant burden. Each item was rated from 0 = *very low or no stress at all* to 4 = *very high stress*. Sum scores were used which could range 0 to 120.

#### Mental health history

Participants were asked at time 1 if they had ever experienced a mental health diagnosis, and this was coded as a binary measure – 0 = no history of diagnoses and 1 = history of diagnoses.

### Data analysis

Associations between breastfeeding variables were measured using chi-square tests. Cross-sectional and longitudinal relationships between pressure to breastfeed at time 1 and mental health outcomes at time 1 and time 2 were examined using linear regression and binary logistic regression. These relationships were also examined when controlling for whether or not someone breastfed, whether or not someone reported experiencing breastfeeding difficulties, and whether or not someone reported a history of a mental health diagnosis. Mental health diagnosis was used as a control rather than the mental health variable at time 1 (e.g., depression) to approximate history of mental health symptoms. Mental health diagnosis would have occurred prior to breastfeeding experiences, but mental health symptoms at time 1 may already reflect reactions to breastfeeding experiences. All analyses were run on SPSS version 29.

Qualitative data on breastfeeding experiences was collected via open ended survey questions from those who reported experiencing pressure to breastfeed, shame about infant feeding choices or stress regarding infant feeding practices. These participants were asked to describe their experiences of pressure, shame and stress. Reflexive thematic analysis techniques were employed on their answers using an experiential approach that aimed to understand participant’s infant feeding experiences (36). We approached our thematic analysis using a critical realist framework, and used a primarily inductive analytic process where codes and themes are developed from data content. We closely followed the 6-step process (37) to construct themes [patterns of shared meaning in the data that are underpinned by a central organizing concept; (38)]. Data was read several times by authors RG and GB, and then the coding process was undertaken by both authors, which involved creating broad descriptive labels for key parts of the text, for example “worry about others opinions.” Following the coding process, initial themes were constructed by RG and GB together by searching for shared meanings or patterns in the codes. RG and GB constructed initial themes separately and then came together to discuss. These themes were then refined by RG and GB, which involved revisiting the codes and full data to determine if the key ideas and experiences were captured by the themes. In terms of positionality, RG is a clinical psychologist and mother of a 3-year-old who she fed during infancy using a combination of breastfeeding, formula and pumped milk due to infant health issues at birth. She has experience working clinically with parents and families in child mental health settings. GB is a clinical psychology trainee who does not have children. Through her training program she has experience working clinically and in a research capacity with children and families.

## Results

In the total sample, 81.3% of mothers reported at time 1 that they breastfed their children (*n* = 183). Of those who did not breastfeed, 14.2% reported this was due to difficulty breastfeeding (*n* = 32), 13.8% reported it was because of their mental health (*n* = 31) and only 3.5% did not because it was easier not to breastfeed (*n* = 8). Of the whole sample, 39.6% reported experiencing strong pressure to breastfeed (*n* = 89), 17.8% reported experiencing shame regarding feeding (*n* = 40), and 51.6% reported experiencing stress due to infant feeding (*n* = 116). Of those experiencing strong pressure to breastfeed, 70.8% reported breastfeeding their baby at time 1 (*n* = 63).

People who did not breastfeed were more likely to indicate they were unable to do their feeding method of choice, *X*^2^(1, *N* = 225) = 39.83, *p* < 0.001, and more likely to report feeling pressure to breastfeed, *X*^2^(1, *N* = 224) = 9.50, *p* = 0.001. Those who did not breastfeed were also more likely to report experiencing shaming for their choice of feeding, *X*^2^(1, *N* = 224) = 12.78, *p* < 0.001. Pressure to breastfeed was not predicted by history of a mental health diagnosis, *X*^2^(1, *N* = 201) = 4.01, *p* = 0.05, but was associated with higher rates of breastfeeding difficulties, *X*^2^(1, *N* = 224) = 22.23, *p* < 0.001. Other key descriptives are presented in Table 1.

**Table 1:** Descriptives of key mental health outcome and control variables.

Outcome or control variables		Mean	SD	Range
Depression	Time 1	8.12	4.65	0 to 22
	Time 2	7.66	4.61	0 to 21
Anxiety	Time 1	13.78	10.4	0 to 53
	Time 2	11.8	9.96	0 to 45
Birth trauma	Time 1	17.39	13.69	0 to 65
	Time 2	15.20	12.85	0 to 66
Stress	Time 1	44.31	17.49	3 to 98
	Time 2	43.15	17.83	5 to 95
		Endorsed by (% of sample)		
Suicide ideation	Time 1	39 (17.3%)		
	Time 2	36 (16.0%)		
Mental health history	Time 1	107 (47.5%)		

### Quantitative results cross-sectional

Strong pressure to breastfeed at time 1 was associated with increased anxiety symptoms [*B* = 5.88, *t* (222) = 4.28, *p* < 0.001], increased depression symptoms [*B* = 1.87, *t* (223) = 3.01, *p* < 0.01], increased birth trauma symptoms [*B* = 6.76, *t* (223) = 3.71, *p* < 0.001], and increased stress [*B* = 9.27, *t* (219) = 3.96, *p* < 0.001], but not suicide ideation (*p* > 0.05) at time 1. These associations remained when controlling for whether or not someone breastfed, whether or not someone experienced difficulties breastfeeding and their mental health history.

### Quantitative results longitudinal

**Figure 1:**
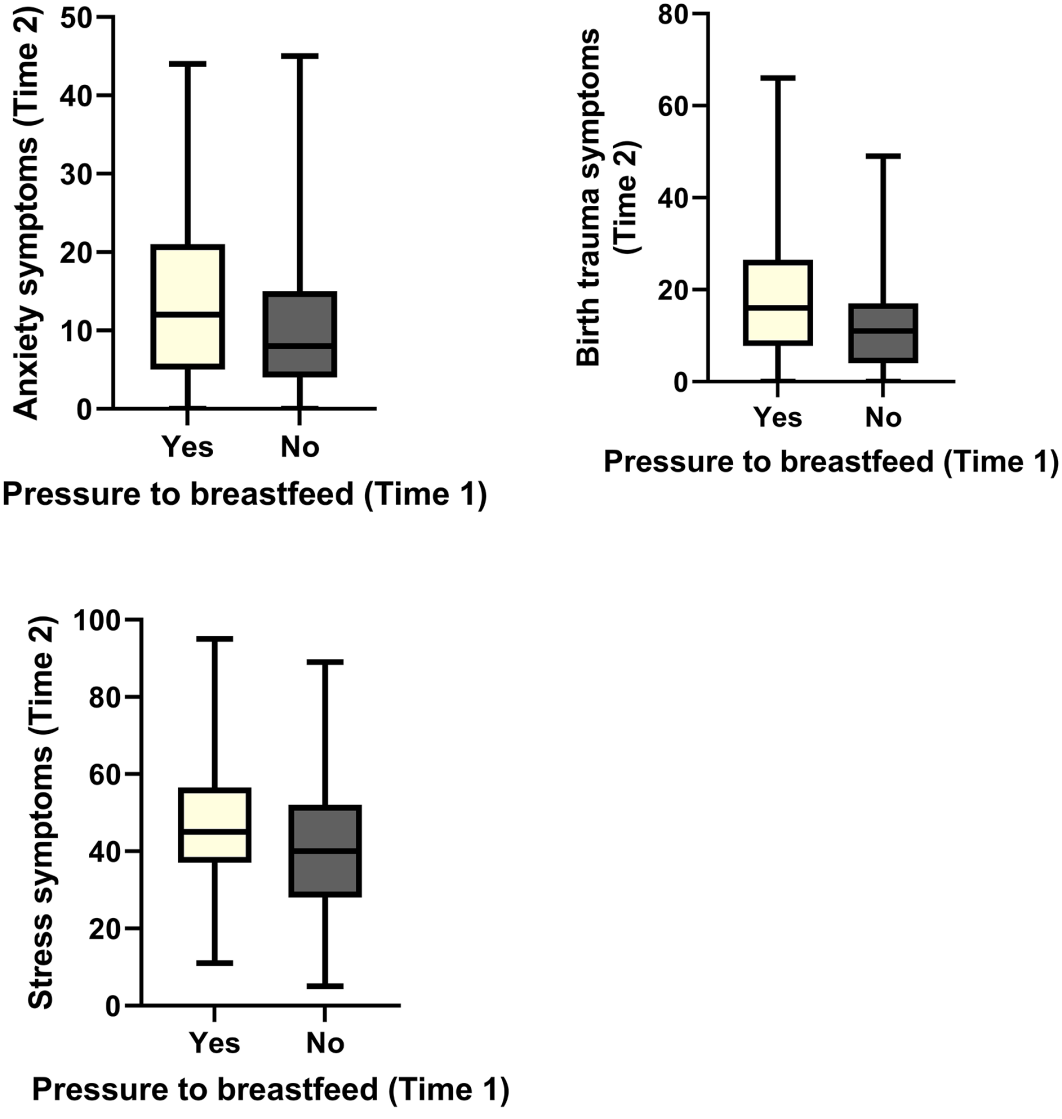
Significant associations between pressure to breastfeed at time 1 and mental health outcomes at time 2.

When examining the longitudinal associations between pressure to breastfeed at time 1 and mental health outcomes at time 2, some relationships remained significant, and others became non-significant. Strong pressure to breastfeed at time 1 was associated with increased anxiety symptoms [*B* = 4.09, *t* (223) = 3.06, *p* < 0.01], increased birth trauma symptoms [*B* = 6.76, *t* (223) = 4.00, *p* < 0.001], and increased stress [*B* = 6.68, *t* (220) = 2.77, *p* < 0.01] at time 2. These associations remained significant when controlling for whether or not someone breastfed, whether or not someone experienced difficulties breastfeeding and their mental health history. This data is shown in Figure 1. Unexpectedly, the relationships between pressure to breastfeed at time 1 and depression and suicide ideation at time 2 were not significant (*p* > 0.05).

### Qualitative results

**Figure 2:**
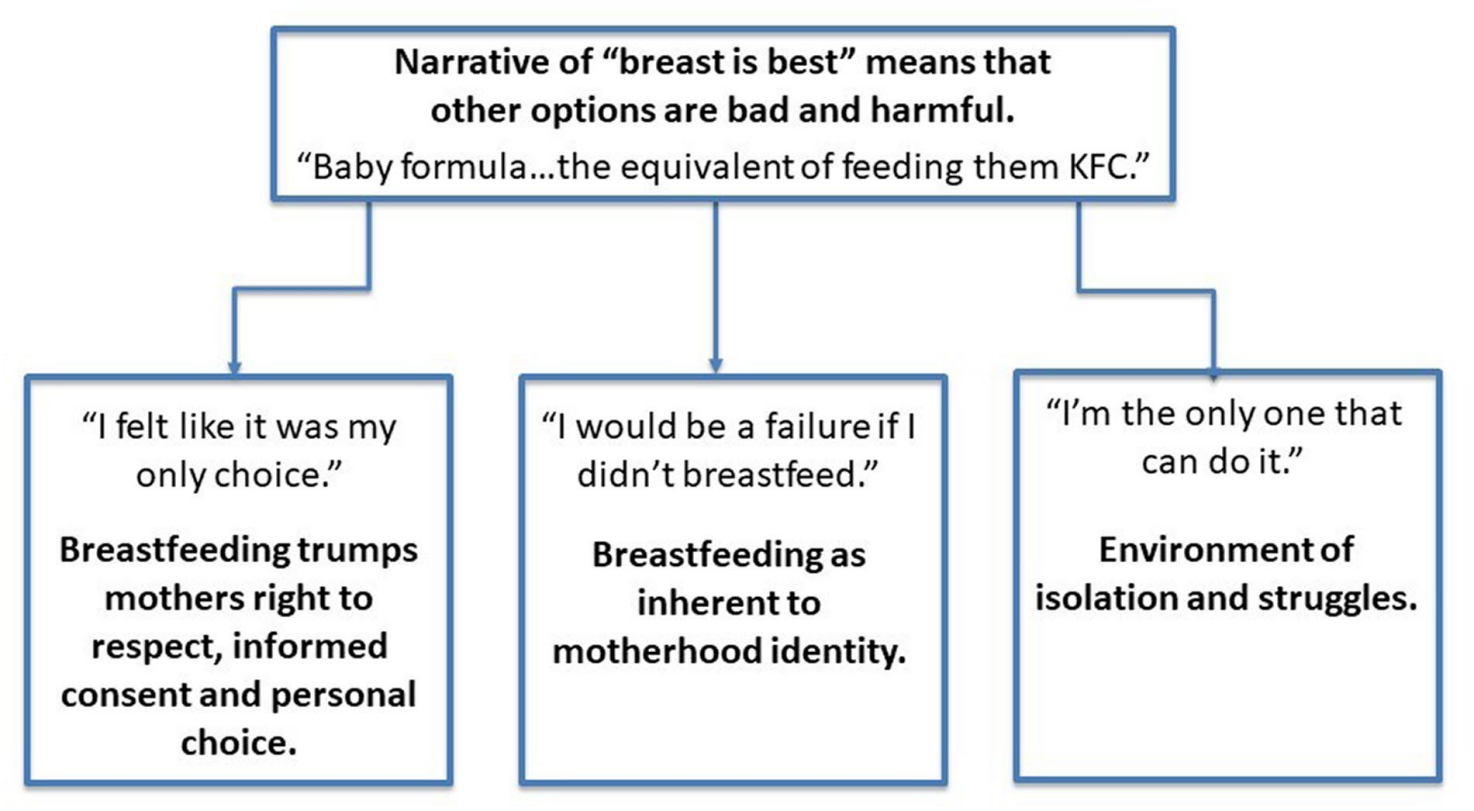
Diagram of qualitative themes.

Those who reported experiencing pressure to breastfeed, shaming around their feeding and/or stress about feeding their infant (*n* = 152) at time 1 were asked to describe these experiences within the survey. From this data, using thematic analysis, we constructed one overarching theme, and three themes that stemmed from the overarching theme. While some participants did report experiencing shame about successful breastfeeding (e.g., people questioning if someone should still be breastfeeding an older child), the overwhelming majority reported shame and pressure related to not breastfeeding, breastfeeding difficulties, and combination feeding. The general structure of our themes is presented in Figure 2, and then these are discussed in more detail.

#### Overarching theme: narrative of breast is best means that other options are bad and harmful

An overarching theme within the data was the shared belief that breastfeeding was the “best” infant feeding option. This belief was often held by the participants themselves, resulting in self-pressure to breastfeed, or it was promoted to participants by their health professionals, family and friends. This belief seemed to be a key factor in the pressure felt by participants to be successful at breastfeeding, as they believed it was the best option for their baby, and they understandably wanted to give their baby the best.

“The usual ‘breast is best’ got brought up a lot especially with the older women in my family.” (Age 32, formula feeding).

“(I was) completely pressured into breastfeeding before, during and post birth. This was my original choice so that was ok.” (Age 37, combination feeding).

Additionally, stemming from this belief was the sentiment that given breastfeeding is the “best” option, this suggests that alternatives to breastfeeding, such as formula feeding, are bad and even harmful for the baby. Again, this idea seemed to originate from a variety of sources including the participants themselves, their friends and family, and the health professionals working with them. This sentiment appeared to promote shame and guilt in those who were unable or not wanting to breastfeed, as it implied they were harming their infants or being selfish.

“My Mum and Mother-in-law comment on formula and how it hurts (infants) tummies.” (Age 34, combination feeding).

“(I was told) formula does not give (infants) the same nutrients.” (Age 33, combination feeding).

“(I) was told by a lactation consultant that giving my baby formula was the equivalent of giving them KFC, even though it was by the order of the doctors.” (Age 28, combination feeding).

“I was told by my midwife if I did not breastfeed I would not bond with my baby and that I was setting him up to get sick much more often.” (Age 29, formula feeding).

#### Theme 2: “I felt like it was my only choice.” Breastfeeding trumps mothers right to respect, informed consent and personal choice

This theme describes a shared experience that many of our participants discussed, in which they felt they were not given a choice in terms of how they fed their infant. Further, many reported they were not given full information about all of the different infant feeding options. For those who were not wanting to breastfeed for a variety of valid reasons, in particular, this lack of choice and autonomy was distressing.

“After the birth of my older child, I never had milk to feed him due to a traumatic delivery and we were readmitted into the hospital 2 days later. The pressure to continue attempting to breastfeed was huge. This time around, I decided from the beginning that I would express only. Many health workers have pressured me to breast feed, even following her birth to just “let her latch.” *I do not feel as though my wishes were listened to.*” (Age 31, Formula feeding and pumping).

“(The) hospital midwife continued to bring up and push breastfeeding at every appointment despite me making it very clear at our first meeting that I did not really want to breastfeed as I had had difficulties with breastfeeding my first baby.” (Age 35, combination feeding).

This feeling of lack of choice appeared to emerge through several different mechanisms including direct pressure to breastfeed applied by health professionals, a lack of alternative feeding options available in hospital, a lack of information about breastfeeding alternatives, and through force (e.g., being denied access to formula or to leaving hospital).

“I wanted to breastfeed but no other option was promoted to us by our midwife or hospital midwives.” (Age 35, exclusive breastfeeding).

“(The hospital) would not have (my) child released from NICU unless I could demonstrate that baby was breastfeeding.” (Age 34, combination feeding).

“In hospital I was even denied (access) when I had asked for some of our formula to be brought (in) as I was mixed feeding.” (Age 28, combination feeding).

“(I) was constantly denied access to formula (in hospital) and was told repeatedly to try expressing.” (Age 24, formula feeding).

#### Theme 3: “I would be a failure if I did not breastfeed.” Breastfeeding as inherent to motherhood identity

This theme describes another belief that came through in the discussion and language used by many of our participants, that suggested they felt breastfeeding was inherent to their identity as a mother, and further than that, they saw breastfeeding as a key element to being a good mother. This was not necessarily a verbalized belief or logical belief that they held, but rather appeared to be a value system that they had internalized. One area where this theme was apparent was in participant language around what it meant about them and their identity if they were having breastfeeding difficulties. Many participants reported either feeling guilt or that they were a “failure” due to breastfeeding difficulties or due to not breastfeeding, or that others had implied that they were a failure due to these reasons and this had induced feelings of guilt or shame.

“(My) mother in law told me I would be a failure if I did not breast feed and need to try harder.” (Age 29, combination feeding).

“When (my) plunket nurse asked (if) I was breastfeeding and I said no she said ‘what not even the colostrum?’. This made me feel very upset and ashamed.” (Age 32, formula feeding).

“I feel guilty if I slip up and use the wrong milk in my tea/consume hidden dairy as it causes bub pain and leads to more sleeplessness.” (Age 30, exclusive breastfeeding).

“I felt disappointed and stressed my body could not produce enough to fully feed my baby.” (Age 34, combination feeding).

Further, participants believed breastfeeding was a natural process and thus should come easy to them. This was another source of guilt and shame for our participants as the discrepancy between their expectation and their experience of breastfeeding was often interpreted as them not doing well as a mother. Similar to the other internalized beliefs and values, these were also put on participants from outside sources.

“(Breastfeeding) did not come naturally to me and I felt very stressed making myself push through to be able to breastfeed.” (Age 27, breastfeeding).

“I kept body shaming myself for not having decent nipples to help my baby latch.” (Age 29, combination feeding).

“(I was told) your eldest is breastfed this one should be easy to do.” (Age 28, exclusive breastfeeding).

#### Theme 4: “I’m the only one that can do it.” Environment of isolation and struggles

This final theme describes that while it was widely acknowledged that breastfeeding was seen as what people should be doing, there was also a common shared experience that it was difficult and isolating. Firstly, participants discussed that due to the nature of breastfeeding being their job, it was a lot of pressure to take on sole responsibility for keeping the baby fed.

“I’m the only one that can do it.” (Age 36, exclusive breastfeeding).

“I ended up being exhausted and stressed after having to breastfeed and express at every single feed so I could provide supply to the father.” (Age 37, combination feeding).

“Breastfeeding is physically demanding, meaning I am more tired and feel overwhelmed sometimes at being the sole source of nutrition for baby.” (Age 30, breastfeeding).

Another element of the isolation was reports that support persons including partners, family and friends could often be unsupportive of the participants’ feeding choices. Participants felt they had to hide their feeding to not make others uncomfortable, or to avoid judgment from others.

“I have to find somewhere discrete to feed her to avoid making other people uncomfortable.” (Age 27, exclusive breastfeeding).

“I felt like I had to be secretive about giving her the occasional formula bottle.” (Age 35, combination feeding).

Finally, most of our participants discussed the huge toll that difficulties breastfeeding or struggles to continue breastfeeding had on their mental health. This seemed to be directly linked with some of the other themes including the link between breastfeeding a being a good mother, in that if the expectations were not met their mental health declined.

“I was in tears every time I needed to feed them. And then I was supposed to go and pump afterwards to build my supply. I could barely eat food or sleep during the first few months, it felt like an impossible task to also breastfeed.” (Age 31, formula feeding).

“Latching was so hard and my mental health was so bad but (I) kept trying to breastfeed.” (Age 25, formula feeding).

## Discussion

Our findings suggest that perceived pressure to breastfeed was associated with concurrent depression, anxiety, stress, and birth trauma symptoms. Pressure to breastfeed was also associated with anxiety, stress, and birth trauma symptoms (but not depression) 4 weeks later. Suicide ideation was not associated with pressure to breastfeed at either timepoint. These relationships were not explained by the positive effects of breastfeeding, breastfeeding difficulties, or a history of mental health difficulties. The key themes we generated from the reported experiences of those who experienced pressure, shame and stress around infant feeding included (1) a discussion that the narrative of “breast is best” was still very present and influential, (2) lacking a choice in this healthcare decision for themselves and their infant, (3) the idea that successful breastfeeding is a key element in one’s identity as a parent and (4) the isolation and stress that breastfeeding and breastfeeding difficulties can bring.

The quantitative findings build on prior qualitative research that suggested feeling pressure to breastfeed can lead to feelings of guilt and shame (23), showing that parents who feel pressure to breastfeed experience higher rates of mental health difficulties concurrently, and 4 weeks later. This difference in mental health symptoms was not explained by this group being less likely to be breastfeeding, which is contrary to other research claiming that breastfeeding promotes better mental health (18). The difference in mental health symptoms for those experiencing pressure to breastfeed was also not explained by higher rates of breastfeeding difficulties in this group. Instead, these results suggest that pressure to achieve optimal infant feeding practices increases anxiety and stress alongside other mental health symptoms such as birth trauma symptoms for parents. This is an important addition to the literature base, which primarily focuses on the possible positive mental health impacts of breastfeeding. It suggests there may be negative mental health outcomes related to breastfeeding practices that extend beyond just experiencing breastfeeding difficulties (39), and that these mental health symptoms may be related to efforts to increase breastfeeding through pressure. It appeared from our qualitative data that some of this increase in mental health symptoms following pressure to breastfeed could be due to a lack of autonomy and choice in decisions made for the health of their infants, the general challenge of successful breastfeeding, alongside judgment from others about decisions not to breastfeed or breastfeeding difficulties. There was also importance placed on breastfeeding being inherent part of motherhood by the participants themselves and also society, that meant when difficulties arose this interpreted as failure as a mother or caregiver.

The qualitative themes and data described in this paper raise some important considerations about how we support mothers and birthing parents when promoting breastfeeding. It appears that at least for some mothers and birthing parents, the current strategies employed in New Zealand, which includes the Baby Friendly Hospital Initiative, can reduce parents’ ability to make informed decisions about their own and their infant’s health. Perhaps in New Zealand a similar dynamic to that noted in Scotland may be occurring, in that this initiative has been interpreted by health-care professionals to indicate they cannot talk about non-breastfeeding feeding options (26). Concerningly, limiting information directly contradicts Health and Disability Consumer Rights in New Zealand, including the right to be fully informed and the right to make an informed choice (Code of Health and Disability Services Consumers’ Rights (40)). Lack of choice and control is also concerning as research shows a lack of control or autonomy during the labor and delivery process can contribute to experiences of birth trauma (41). In addition, there were reports of non-scientific or scaremongering information being shared, perhaps with the intention of encouraging people to breastfeed. For example, that formula is equivalent to KFC, and that formula feeding parents are unable to bond with their infants. This type of breastfeeding intervention is not new, and has been seen internationally (42). This type of care is likely to increase feelings of anxiety and shame, and again would violate Health and Disability Consumer Rights in New Zealand (Code of Health and Disability Services Consumers’ Rights (40)), and likely similar laws internationally. A perceived lack of control on top of the increased mental health difficulties experienced by those who reported perceived pressure to breastfeed indicates an increased need for autonomy and support in our postpartum health interventions.

Other qualitative themes from this analysis spoke to the breastfeeding ideals that are promoted in (certain) societies, which are likely to contribute to personal ideals about motherhood and parenthood. For example, the idea that women and birthing parents cannot be good or successful mothers and parents if they do not breastfeed, or if they breastfeed less than the desired amount of time. Such ideals around breastfeeding are likely to be determined by the society and culture one is living in, with research in developing countries suggesting there are beliefs about practices that might be less conducive to breastfeeding such as offering other foods to infants, and community pressure to discontinue breastfeeding (43). In the New Zealand population where our study was conducted, the views reported primarily supported the idea “breast is best,” although one or two participants reported being pressured to formula feed or not breastfeed past a certain age. These types of ideals, although they may appear positive given they support the breastfeeding ideals of health professionals, can be unhelpful as they lack nuance around the complexity of issues such as infant feeding. Concrete ideals or beliefs can contribute to an unhealthy mental perspective, as we can never reach perfection all the time (44), and other unrealistic maternal expectations have been shown to be associated with depression (45). Thus, these ideals may contribute to the increased rates of mental health difficulties in those reporting perceived pressure to breastfeed.

Our qualitative themes aligned with other qualitative work in culturally similar countries such as England, that suggested mothers were mostly aware breastfeeding was “best” for their baby, and most women wanted to successfully breastfeed (46). It also supported findings that suggest guilt is a big issue for women who struggle with breastfeeding (46). The present findings also supported the idea that social support is important for successful breastfeeding, as our participants talked about how hard it was being the only one doing the feeding (47). Another concept that has come up in prior work about breastfeeding is that health professionals should consider more honesty upfront about breastfeeding difficulties (47), and it seems possible that this could be a helpful technique to support a more balanced discussion of infant feeding options, alongside reducing guilt when experiencing breastfeeding difficulties. Given guilt is linked to increased mental health difficulties (48), this technique may also assist with reducing rates of mental health symptoms.

Unexpectedly, the impact of perceived pressure to breastfeed on depression symptoms was not apparent 4 weeks later, and there was no relationship between pressure to breastfeed and suicide ideation. This suggests the longer-term impacts of perceived pressure to breastfeed may be specific to certain types of mental health symptoms, rather than exacerbating all types of distress more generally. A possible contributing mechanism to the relationship between perceived pressure to breastfeed and mental health symptoms is lack of control. As shown in our qualitative analysis, many experiencing pressure to breastfeed felt they lacked decision making power or choice in their feeding decisions, which would result in reduced feelings of control. Control plays an important role in the development of anxiety (49), stress (50) and trauma (51), but is implicated less in the development of depression and suicide ideation, and so is one possible explanation for the differential impacts on types of mental health symptoms.

These findings must be considered in light of methodological limitations. The sampling procedure utilized involved any interested participants responding to an advertisement about postpartum mental health. As such, the included participant group may involve higher rates of people with mental health difficulties or those who are interested in mental health when compared to the general population. Further, a large section of our original sample were excluded due to not completing the survey, and this may bias the final sample, as those who are less able to complete the survey may be experiencing higher rates of stressors or mental health difficulties that make participating fully more difficult. The participants are also all from New Zealand, and are primarily New Zealand European, and thus are likely to experience a different culture around breastfeeding than other ethnic groups or people from other countries [e.g., (43)]. These findings would be strengthened by replications in more diverse populations, and using additional sampling strategies. Finally, the study focuses on self-reported breastfeeding pressure, and it is possible that perceived pressure to breastfeed differs from actual experienced pressure to breastfeed, and may be influenced by mental health symptoms. Future research could follow participants from earlier on in their pregnancy and breastfeeding journey to gain a more nuanced understanding of this longitudinal relationship.

These mixed methods findings suggest that increasing pressure to breastfeed through interventions is likely to be detrimental to maternal mental health, and as we know, poor maternal mental health does not promote likelihood of exclusive breastfeeding (52) or prolonged breastfeeding (16). Of course we must also acknowledge some perceived pressure is internal to the person, and not applied from external sources. However, we can also acknowledge that significant pressure is being applied from external sources as part of many breastfeeding interventions. So then, given this information, and acknowledging the positive effects of breastfeeding, how do we approach breastfeeding promotion? We have come up with the following three recommendations based on our quantitative and qualitative data. These are discussed in relation to health laws in New Zealand, but are also relevant to international settings.

1.We need to provide balanced scientific information on all feeding practices.

In New Zealand health consumers are protected by the Health and Disability Services Consumers’ Rights, and one of these rights is the right to be fully informed. Many of our participants indicated they were only educated on breastfeeding practices. This directly goes against their right to be fully informed, and may be a result of incorrect interpretations of the Baby-Friendly Hospital Initiative (26). This indicates that in maternal and infant health settings there may not be as much emphasis on patient centered care and informed choice, but rather more emphasis placed on paternalistic medical models (53). It appears from our data that a lack of input into the decision-making process, and a lack of respect for parental decisions contributes to negative mental health outcomes. As such we suggest that health-care professionals provide parents with all feeding options, alongside accurate scientific information, displayed in a readable and digestible format, about the pros and cons of each approach and that parents be allowed to make their own informed choices. This information should include discussion of breastfeeding challenges (9). If we are confident breastfeeding is the best choice for all infants, we should have no concerns the majority of parents will come to the same conclusion while being presented with all of the evidence.

2.Informed feeding choices should be respected and supported by all health professionals.

Many parents who experienced pressure to breastfeed reported that their choices were not respected. Another right under the Health and Disability Services Consumers’ Rights in New Zealand is the right to make an informed choice, and so again this is an unacceptable practice that reverts to paternalistic medical models. Lack of choice is highly likely to contribute to feelings of lack of control, which is associated with anxiety, stress and trauma as discussed above. By respecting parents’ choices and supporting them on their feeding journeys, we are more likely to be supporting mentally well parents who can provide an optimal environment at home for infant development. Policy makers may want to consider this right to choice, and the role of shame and guilt when considering current laws such as those preventing discussion and education of bottle feeding in certain settings.

3.Individualized breastfeeding support in particular, should be the intervention of choice.

To increase rates of breastfeeding it is clear interventions should aim to decrease stress, and increase supports. True support should involve an individualized approach where we listen to the individual and their unique needs (3). Some factors that could be considered by health-care professionals in an individualized approach include: What are their personal concerns or worries about the process, and how can we support them with these? What difficulties are they experiencing, and how can we help them with these? What additional needs do they have to reach their feeding goals? The actual support itself might look like lactation consultants, as is the typical support approach (when breastfeeding support is offered), but depending on the individual and their particular concerns it might also look like empathy and normalization, reassurance, mental health support, social support groups, and/or supporting them with feeding supplementation efforts. There is typically a hesitation to offer feeding supplementation due to risk of reduced milk supply, however again we need to consider the person’s right to have all the information and to choose what is best for themselves and their infant. Many people successfully combination feed long-term. In a large cohort study, parents who were combination-feeding breastfed for shorter periods of time than those exclusively breastfeeding, however at 4 months 65% of the exclusive breastfeeding group were still breastfeeding, and 40% of the combination-feeding group were still breastfeeding (54). If an additional benefit of combination feeding is improved mental health and reduced pressure on the mother, positive effects of this may on the child may outweigh any potential negative impact of reduced intake of breastmilk. Further, one of the most common reasons mothers cease breastfeeding is perceived lack of supply (55). While for some of those mothers, improved lactation support might improve this issue, combination-feeding might provide another acceptable alternate option to ceasing breastfeeding. Promotion of a wider range of options for breastfeeding difficulties could be considered by health-care policy makers to support these ideas. Given social support is often identified as a key element to successful breastfeeding (56), interventions should consider how to increase wider supports as well. This might be through wider societal changes, for example not only offering paid leave for the birthing parent, but also offering paid partner leave that would allow the partner of the breastfeeding parent to be at home and offering support. It could also involve health-care professionals encouraging support people to attend lactation support sessions, so they can also be providing guidance or other supports when needed. Finally work-places should also consider their role in supporting mothers feeding choices, and provide flexibility in return-to-work procedures enabling mothers to structure their lives in a way that may allow a return to work and successful feeding practices for their infants.

In conclusion, we may be doing more harm than good by focusing our interventions for breastfeeding primarily on increasing pressure to breastfeed, and by presenting breastfeeding as the only viable option, we may be at risk of violating the code of Health and Disability Services Consumers’ Rights in New Zealand. This research indicates that pressure to breastfeed likely has negative impacts on maternal mental health, particularly for those who place a high importance on breastfeeding as the role of a successful mother. This pressure may also stem from a lack of choice and autonomy in healthcare settings, which increases guilt and shame. Given increased stress and reduced maternal mental health is associated with reduced exclusive breastfeeding and breastfeeding duration, we should instead be offering supportive intervention approaches that include the supply of balanced evidence, where all informed choices are supported. This approach is likely to increase breastfeeding rates, improve child development and improve maternal mental health. In support of these ideas, one of the few interventions with good support for increasing breastfeeding are counseling interventions which include a support element (28, 57).

## Data availability statement

The raw data supporting the conclusions of this article will be made available by the authors, without undue reservation.

## Ethics statement

The studies involving humans were approved by Victoria University of Wellington Human Ethics Committee (#30643). The studies were conducted in accordance with the local legislation and institutional requirements. The participants provided their written informed consent to participate in this study.

## Author contributions

RG: Conceptualization, Data curation, Formal analysis, Funding acquisition, Investigation, Methodology, Project administration, Resources, Software, Supervision, Validation, Visualization, Writing – original draft, Writing – review & editing. SL: Conceptualization, Data curation, Project administration, Writing – review & editing. GB: Conceptualization, Methodology, Project administration, Writing – review & editing.
